# Structural and functional analysis of the small GTPase ARF1 reveals a pivotal role of its GTP-binding domain in controlling of the generation of viral inclusion bodies and replication of grass carp reovirus

**DOI:** 10.3389/fimmu.2022.956587

**Published:** 2022-08-26

**Authors:** Jie Zhang, Pengwei Li, Riye Lu, Songying Ouyang, Ming Xian Chang

**Affiliations:** ^1^ State Key Laboratory of Freshwater Ecology and Biotechnology, Institute of Hydrobiology, Chinese Academy of Sciences, Wuhan, China; ^2^ College of Advanced Agricultural Sciences, University of Chinese Academy of Sciences, Beijing, China; ^3^ Provincial University Key Laboratory of Cellular Stress Response and Metabolic Regulation, the Key Laboratory of Innate Immune Biology of Fujian Province, Biomedical Research Center of South China, Key Laboratory of OptoElectronic Science and Technology for Medicine of the Ministry of Education, College of Life Sciences, Fujian Normal University, Fuzhou, China; ^4^ Innovation Academy for Seed Design, Chinese Academy of Sciences, Wuhan, China

**Keywords:** grass carp reovirus, viral inclusion bodies, nonstructural proteins NS80 and NS38, ADP ribosylation factor 1, gcARF1-GDP complex, GTPase activity

## Abstract

Grass carp reovirus (GCRV) is the most pathogenic double-stranded (ds) RNA virus among the isolated aquareoviruses. The molecular mechanisms by which GCRV utilizes host factors to generate its infectious compartments beneficial for viral replication and infection are poorly understood. Here, we discovered that the grass carp ADP ribosylation factor 1 (gcARF1) was required for GCRV replication since the knockdown of gcARF1 by siRNA or inhibiting its GTPase activity by treatment with brefeldin A (BFA) significantly impaired the yield of infectious viral progeny. GCRV infection recruited gcARF1 into viral inclusion bodies (VIBs) by its nonstructural proteins NS80 and NS38. The small_GTP domain of gcARF1 was confirmed to be crucial for promoting GCRV replication and infection, and the number of VIBs reduced significantly by the inhibition of gcARF1 GTPase activity. The analysis of gcARF1-GDP complex crystal structure revealed that the ^27^AAGKTT^32^ motif and eight amino acid residues (A^27^, G^29^, K^30^, T^31^, T^32^, N^126^, D^129^ and A^160^), which were located mainly within the GTP-binding domain of gcARF1, were crucial for the binding of gcARF1 with GDP. Furthermore, the ^27^AAGKTT^32^ motif and the amino acid residue T^31^ of gcARF1 were indispensable for the function of gcARF1 in promoting GCRV replication and infection. Taken together, it is demonstrated that the GTPase activity of gcARF1 is required for efficient replication of GCRV and that host GTPase ARF1 is closely related with the generation of VIBs.

## Highlights

1. gcARF1 is indispensable for the formation of VIBs during GCRV infection.
2. GCRV NS80 and NS38 proteins can interact with gcARF1 and recruit gcARF1 into VIBs.
3. The amino acid residues (27AAGKTT32, N126, D129, A160) of gcARF1 are essential for GDP binding and GCRV replication.

## Introduction

The ADP ribosylation factors (ARF) belong to the Ras superfamily of small GTPases, which are guanine-nucleotide dependent molecular switches involved in regulating of numerous cellular processes (1). Based on amino-acid sequence identity, mammalian ARF proteins can be divided into 3 classes: ARF1 and ARF3 (class I), ARF4 and ARF5 (class II) and ARF6 (class III) (2). The amino-acid sequences of ARF proteins are well conserved in all eukaryotes, from yeast to humans (3). Like other small GTPases, ARF proteins cycle between their inactive and active conformations, which is achieved by exchanging GTP for GDP *via* guanine nucleotide exchange factors (GEFs) to form active-GTP-bound form and then hydrolyzing GTP to switch back to inactive-GDP-bound form *via* GTPase activating proteins (GAPs) (3–5). ARF proteins play a key role in membrane traffic, mitochondrial architecture, assembly and dynamics of the microtubule and actin cytoskeletons (3, 6).

Among mammalian ARF proteins, ARF1 is a well-studied member, and has a well-established role in the assembly and budding of Golgi coat proteins coatomer (COPI) vesicles at the Golgi (7). Recent research has shown that ARF1 has a role in viral replication and infection. ARF GTPases are required for different steps of cytomegalovirus infection, and the knockdown of ARF1 can abolish the establishment of cytomegalovirus infection (5). During enterovirus infection, ARF1 was recruited to the replication organelles, and co-localized with the viral nonstructural protein 2B and mature virions. Different from other class I and II ARF proteins, only the depletion of ARF1 significantly increased the sensitivity of enterovirus replication to brefeldin A (BFA), a potent inhibitor of viral replication such as many (+) RNA viruses including enteroviruses (8). For hazara nairovirus (HAZV), its replication cycle can be divided into at least two distinct phases. The second phase involved in infectious virus production is highly COPI- and ARF1- dependent (8). For aquatic animal viruses, a study showed that knockdown of ARF1 from giant freshwater prawn *Macrobrachium rosenbergii* decreased the replication level of white spot syndrome virus (9). However the mechanism that ARF1 is involved in the infection and replication of aquatic animal viruses remains unclear.

Grass carp reovirus (GCRV) is recognized as the most pathogenic among the isolated aquareoviruses, which contains a genome of 11 double-stranded (ds) RNA segments enclosed in a core surrounded with a double layered icosahedral capsid (10). The 11 genomic segments encode five nonstructural proteins (NS80, NS38, NS31, NS26 and NS16) and seven structural proteins (VP1 to VP7) (11). Previous review has summarized the known GCRV strains and antiviral immune responses of high-mobility group box proteins (HMGBs), TLRs, RLRs and NLRs signaling pathways in response to GCRV infection (12). Furthermore, it is found that the replication and assembly of GCRV take place in specific intracellular compartments called viral inclusion bodies (VIBs) (13). The nonstructural proteins NS80 and NS38 of GCRV are two main proteins to form the VIBs, and can recruit viral and host factors into VIBs to assist the replication and assembly of GCRV (14–16). Whether viral infections from GCRV recruit ARF proteins to cytoplasmic VIBs remains unclear. Here, we found that grass carp ARF1 (gcARF1) can promote GCRV replication and infection, which is dependent on the GTPase activity of ARF1. The nonstructural proteins NS80 and NS38 of GCRV can interact with the small_GTP domain of gcARF1, and recruit gcARF1 into cytoplasmic VIBs. When the GBF1-mediated activation of ARF1 is blocked by BFA (17), the number of VIBs produced during GCRV infection is significantly reduced. We also resolved the crystal structure of gcARF1 protein, and found the ^27^AAGKTT^32^ motif and eight amino acid residues (A^27^, G^29^, K^30^, T^31^, T^32^, N^126^, D^129^ and A^160^) of gcARF1 necessary for binding to GDP. Furthermore, the ^27^AAGKTT^32^ motif and the amino acid residue T^31^ of gcARF1 are crucially important for promoting replication and infection of GCRV. Our study thus reveals a new critical function for gcARF1 in generation of VIBs.

## Materials and methods

### Cells, virus and plasmids

CIK (*Ctenopharyngodon idellus* kidney) cells were grown in minimum essential medium (MEM) supplemented with 10% FBS. Grass carp reovirus (GCRV-873) was propagated in CIK cells using MEM supplemented with 2% FBS. Plasmids used in this study including pTurboGFP vector (Evrogen), p3×FLAG-CMV™-14 Expression Vector (Sigma-Aldrich Co. LLC), and pET28a-SUMO vector were previously prepared and stored in our laboratory. The GenBank accession numbers of gcARF1 is OM567585. gcARF1-GFP was obtained using the primer pairs gcARF1F1/gcARF1R1 and cloned into the pTurboGFP-N vector. gcARF1-FLAG, gcARF1-small_GTP-FLAG, gcARF1(d27-32aa)-FLAG, and gcARF1(T31N)-FLAG were obtained using the primer pairs gcARF1F/gcARF1R, gcARF1-small_GTPF/gcARF1-small_GTPR, gcARF1(d27-32aa)F/gcARF1(d27-32aa)R, and gcARF1(T31N)F/gcARF1(T31N)R, and cloned into the p3×FLAG-CMV-14 vector, respectively. pET28a-gcARF1 was obtained using the primer pairs gcARF1F2/gcARF1R2, and cloned into the pET28a-SUMO vector. The primers used for plasmid constructs are listed in **Table S1**.

### Antibodies and reagents

The anti-FLAG mouse monoclonal antibody (#F3165), anti-pTurboGFP rabbit polyclonal antibody (#AB513) and anti-GAPDH mouse monoclonal antibody (#60004-1-Ig) were purchased from Sigma-Aldrich, Everogen and Proteintech, respectively. To obtain these antibodies against NS80, NS38, VP3 and VP5 proteins of GCRV, the 2~160 amino acids (aa) of NS38, 500~692 aa of NS80, 2~200 aa of VP3 or 451~ 648 aa of VP5 was inserted into the pET-32a (+) vector (EMD Millipore) for prokaryotic expression. Purified recombinant proteins were used to immunize Japanese White rabbits to acquire the rabbit polyclonal antibodies and mice to acquire the mouse polyclonal antibodies. The antiserum from the rabbit was affinity-purified on antigen-coupled CNBr-activated agarose (GE Healthcare). Goat-anti-mouse Ig-HRP conjugate secondary antibody, Goat-anti-rabbit Ig-HRP conjugate secondary antibody, Alexa Fluor 488-conjugated secondary Ab against mouse IgG, Alexa Fluor 594-conjugated secondary Ab against rabbit IgG, 6-diamidino-2-phenylindole (DAPI), Lipofectamine 2000 and Protease inhibitor cocktail were purchased from Thermo Fisher Scientific. Dimethylsulfoxide (DMSO) and the FLAG^®^ Immunoprecipitation Kit was purchased from Sigma-Aldrich. Brefeldin A (BFA, S7046) was purchased from Selleck. Golgi-tracker red (C1043) was purchased from Beyotime.

### Knockdown of gcARF1 by siRNA

Transient knockdown of gcARF1 was achieved by transfection of siRNA targeting gcARF1 mRNA. Three siRNA sequences including siARF1-1 (5′-CGTCACTACTTCCAGAACA-3′), siARF1-2 (5′-GCAGGCAAGAGCTTCTTTA-3′) and siARF1-3 (5′-GCAATGAATGCTGCAGAAA-3′) targeting different regions of gcARF1 were synthesized by RIBOBIO (Guangzhou, China). CIK cells were transfected with siRNA using Lipo 2000 for 24 h. Silencing efficiencies of these three siRNAs were evaluated by qRT-PCR, and results were compared with the control-siRNA provided by the supplier. A preliminary experiment indicated that siARF1-3 possessed the best silencing efficiency at a final concentration of 100 nM, and used for the present study.

### Viral infection assays

To investigate the effects of gcARF1 or its mutants in GCRV infection, CIK cells grown in 12-well plates were transfected with 1000 ng FLAG empty plasmid, gcARF1-FLAG, gcARF1-small_GTP-FLAG, gcARF1(d27-32aa)-FLAG, or gcARF1(T31N)-FLAG respectively. For the effects of gcARF1 knockdown in GCRV infection, CIK cells grown in 12-well plates were transfected with the control-siRNA or siARF1. After 24 h post-transfection, cells were infected with GCRV at an MOI of 1 in serum-free MEM medium at 25°C for 1 h. Following adsorption, cells were washed with PBS to remove non-adsorbed virions. Then, the infected cells were maintained in 2% FBS MEM at 25°C for 24 h.

For inhibition of gcARF1 GTPase activity by BFA, CIK cells were plated in 24-well or 6-well plates, and then the growth medium was replaced with the same medium supplemented with 0.5 μg/ml, 2.5 μg/ml or 10 μg/ml of BFA (stored as a 10 mM/mL stock in DMSO at -80°C), or the equivalent volume of DMSO alone as a control. For BFA pretreatment before GCRV infection, CIK cells were incubated with or without BFA for 1 h, then washed with PBS to remove BFA, and finally infected with GCRV for 1 h. For BFA treatment during virus attachment, CIK cells were infected with GCRV and treated with BFA for 1 h, and then washed with PBS to remove BFA and non-adsorbed virions. For BFA treatment after virus attachment, CIK cells were infected with GCRV for 1 h, then washed with PBS to remove non-adsorbed virions, and finally treated with BFA for another 24 h. For BFA treatment during virus attachment and after virus attachment, CIK cells were infected with GCRV and treated with BFA for 1 h, then washed with PBS to remove BFA and non-adsorbed virions, and finally treated with BFA for another 24 h.

The cells without BFA treatment and/or GCRV infection were used for the control group. The culture supernatants of infected cells were collected for determination of GCRV titers. The cells in the 24-well plates were used for crystal violet staining. The cells in the 6-well plates were used for protein extraction and Western Blotting.

### RNA extraction, reverse transcription and qRT-PCR

For the overexpression of gcARF1, CIK cells seeded overnight in 6-well plates at 1×10^6^ cells per well were transiently transfected with 1000 ng FLAG, or gcARF1-FLAG (500 ng or 1000 ng). For the knockdown of gcARF1, CIK cells seeded overnight in 6-well plates at 1×10^6^ cells per well were transiently transfected with the control-siRNA or siARF1. At 24 h after transfection, these cells were infected with GCRV at an MOI of 1. Then these cells were collected at 24 hpi, and used for RNA extraction using TRIzol Reagent (Thermo Fisher Scientific). The concentration of total RNA was determined by using the spectrophotometer (NanoDrop 2000; Thermo). RNase-free DNase I (Thermo) was used to remove genomic DNA remnants at 37°C for 30 min. The cDNA was synthesized using the RevertAid™ First Strand cDNA Synthesis Kit (Thermo Fisher Scientific) according to the manufacturer’s instructions. qRT-PCR was performed on a BIO-RAD CFX96™ C1000 thermal cycler using iQ™ SYBR Green Supermix (BioRad, Singapore) under the following conditions: 3 min at 95°C, followed by 45 cycles of 10 s at 95°C, 15 s at 60°C and 10 s at 72°C. All reactions were performed in triplicate and the mean value recorded. Those GCRV genes including NS80, NS38, VP1, VP2, VP3, VP4, VP5, VP6, and VP7 were used for qRT-PCR. The housekeeping genes including β-actin, EF-1α and 18S rRNA were used for normalizing cDNA amounts. The fold changes relative to the control group transfected with the FLAG empty plasmid or control-siRNA were calculated using the 2^-ΔΔCt^ method. All primers used for qRT-PCR are shown in **Table S1.**

### Protein purification of gcARF1

The full-length of gcARF1 was cloned from grass carp and inserted into pET28a expression vector. The constructed pET28a-gcARF1 plasmid was transformed into *E. coli* BL21 (DE3) cells. The cells were grown in LB medium at 37°C with constant shaking at 220 rpm about 2.5 h and induced with 0.3 mM isopropyl-b-D-thiogalactopyranoside (IPTG) when the bacteria grew to a density OD_600_ (optical density at 600 nm) = 1.0. The bacteria were cultured for 16 h at 16°C, pelleted by centrifugation, and resuspended in the cold lysis buffer (50 mM Tris-HCl, pH 7.5, 150 mM NaCl). Following lysis by ultrasonication, the cell lysates were centrifuged at 17000 rpm for 30 min at 4°C. The protein with His-SUMO tag was purified by affinity chromatography (Ni ^2+^ resin). The His-SUMO tag was removed by SUMO Protease ULP1. The tag-free protein was purified by size-exclusion chromatography using a Superdex 200 Increase column (GE Healthcare) equilibrated with buffer containing 25 mM HEPES, 150 mM NaCl and 2 mM DTT. The purified protein was finally collected and concentrated to A_280_ = 15 for crystallization screen.

### Crystallization, data collection and structure determination

Crystallization screens were performed using the hanging-drop vapor diffusion method at 16°C, with drops containing 0.5 μl of protein solution mixed with 0.5 μl of reservoir solution. Diffraction quality of gcARF1 crystals was obtained 0.1 M Sodium citrate tribasic dihydrate, pH 5.5, 22% polyethylene glycol 1000. Crystals were harvested and flash-frozen in liquid nitrogen with the 20% ethylene glycol as a cryoprotectant. Complete X-ray diffraction datasets were collected at BL02U1 beamline of Shanghai Synchrotron Radiation Facility (SSRF). Diffraction images were processed with HKL-200 program. Crystal tructure of gcARF1 was solved by molecular replacement (MR) using Mus musculus ADP-ribosylation factor-like protein 3 as a model (PDB code: 3BH7). Model building and crystallographic refinement were carried out in Coot v0.8.2 and PHENIX v1.10.1 (18, 19). The interactions were analyzed with PyMOL (http://www.pymol.org/), PDBsum and LigPlus. The Figures were generated in PyMOL. The accession number for gcARF1-GDP complex reported in this paper is PDB ID 7WQY.

### Immunofluorescence assays

To determine the possible co-localization of gcARF1 with VP3, VP5, NS38 or NS80 of GCRV, CIK cells were plated onto coverslips in 24-well plates, and then transfected with gcARF1-FLAG. After 24 h, CIK cells were infected with GCRV or left untreated. At 24 hpi, the cells were washed twice with PBS and fixed with 4% PFA for 1 h. After being washed three times with PBS, the cells were incubated with anti-FLAG (1:1000), rabbit anti-NS80 or anti-VP5 (1:500) Ab, or mouse anti-NS38 or anti-VP3 (1:500) Ab, followed by incubation with Alexa Fluor 488-conjugated secondary Ab against mouse IgG (1:400) or Alexa Fluor 594-conjugated secondary Ab against rabbit IgG (1:400).

To determine the numbers of VIBs during GCRV infection with or without the BFA treatment, CIK cells were plated onto coverslips in 24-well plates, and then infected with GCRV for 1 h or left untreated. The cells were washed with PBS to remove non-adsorbed virions. Then, the infected cells were maintained in 2% FBS MEM supplemented with 2.5 μg/ml of BFA, or the equivalent volume of DMSO alone as a control at 25°C for 24 h. At 24 hpi, the cells were washed twice with PBS and fixed with 4% PFA for 1 h. After being washed three times with PBS, the cells were incubated with rabbit anti-NS80 or anti-VP5 (1:500) Ab, or mouse anti-NS38 (1:500) Ab, followed by incubation with Alexa Fluor 488-conjugated secondary Ab against mouse IgG (1:400) or Alexa Fluor 594-conjugated secondary Ab against rabbit IgG (1:400). DAPI staining was applied to detect the cell nucleus. After each incubation step, cells were washed with PBS. Finally, the coverslips were washed and the images were obtained using a SP8 Leica laser confocal microscopy imaging system.

To determine the effects of the depletion of ^27^AAGKTT^32^ motif or the mutation of T31 residue on the formation and generation of VIBs during GCRV infection, CIK cells were plated onto coverslips in 24-well plates, and then transfected with FLAG, gcARF1-FLAG, gcARF1(d27-32aa)-FLAG or gcARF1(T31N)-FLAG, respectively. After 24 h post-transfection, the cells were infected with GCRV at an MOI of 1 and maintained in MEM containing 2% FBS. At 24 hpi, the cells were washed twice with PBS and fixed with 4% PFA for 1 h. After being washed three times with PBS, the cells were incubated with anti-FLAG (1:1000) and rabbit anti-NS80, followed by incubation with Alexa Fluor 488-conjugated secondary Ab against mouse IgG (1:400) and Alexa Fluor 594-conjugated secondary Ab against rabbit IgG (1:400). DAPI staining was applied to detect the cell nucleus. After each incubation step, cells were washed with PBS. Finally, the coverslips were washed and the images were obtained using a SP8 Leica laser confocal microscopy imaging system. The Image J was used to detect the mean fluorescence intensity of VIBs.

To investigate the effect of BFA on distribution of Golgi apparatus in the presence and absence of GCRV infection, CIK cells plated in 24-well plates were infected with GCRV for 1 h or left untreated. The cells were washed with PBS to remove non-adsorbed virions. Then, the cells were maintained in 2% FBS MEM supplemented with 2.5 μg/ml of BFA or the equivalent volume of DMSO as a control at 25°C for 24 h. The cells were washed with PBS and incubated with Golgi-Tracker Red at 4°C for 30 min. DAPI staining was applied to detect the cell nucleus. Finally, the images were obtained using a SP8 Leica laser confocal microscopy imaging system.

### Co-immunoprecipitation assay and western blotting

CIK cells seeded in 10-cm^2^ dishes were transfected with various indicated plasmids. After 24 h post-transfection, the cells were infected with GCRV at an MOI of 1 and maintained in 2% FBS MEM at 25°C for another 24 h. Then, the cells were lysed in 600 μl IP lysis buffer containing protease inhibitor cocktail. Cellular debris was removed by centrifugation at 12,000 × g for 10 min at 4°C. Co-IP was performed using the FLAG-tagged Protein Immunoprecipitation Kit according to the manufacturer’s manual. The agarose was washed six times with ice-cold washing solution, and protein was eluted with Elution Buffer.

For Western blotting analysis, the whole-cell extracts were subjected to 10% SDS-PAGE and transferred to PVDF membranes, followed by blocking with 5% nonfat milk in Tris-buffered saline-Tween (TBST) for 1 h. The membrane was washed, and then incubated with primary antibody (Ab) overnight at 4°C. The primary Abs including anti-GAPDH (1: 5000), anti-FLAG (1: 5000), anti-pTurboGFP (1: 5000), anti-NS80 (1: 5000), anti-NS38 (1: 5000), anti-VP3 (1: 5000), or anti-VP5 (1: 5000) were used. After washing with TBST, the membrane was next incubated with Goat-anti-mouse Ig-HRP conjugate secondary Ab (1: 5000) or Goat-anti-rabbit Ig-HRP conjugate secondary Ab (1: 5000) for 1 h at room temperature. The bands were detected using Pierce ECL Western Blotting Substrate and ECL Western blot system (LAS-4000mini).

### Statistical analysis

Statistical analysis and graphs were performed and produced using Graphpad Prism 7.0 software. Data are presented as mean and SEM. The significance of results was analyzed by Student’s t-test and one-way analysis of variance with Bonferroni correction (*p < 0.05, **p < 0.01).

## Results

### ARF1 promotes GCRV replication and infection

**Figure 1:**
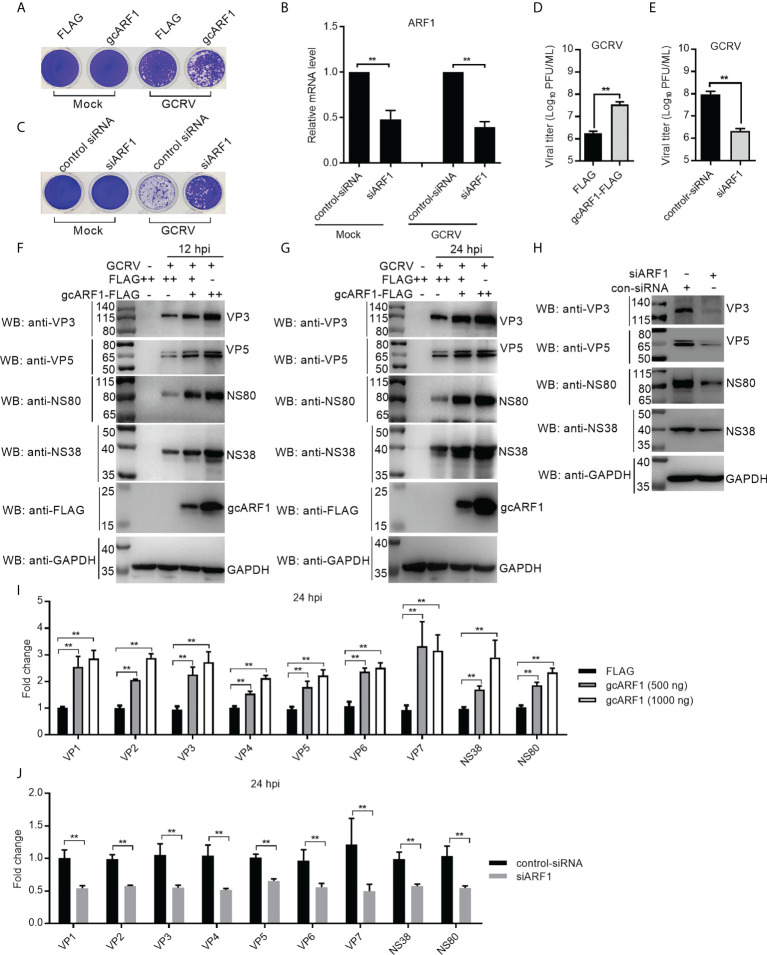
gcARF1 promotes GCRV infection. **(A)** Crystal violet staining for overexpression of gcARF1 in CIK cells that were mock infected or infected with GCRV at an MOI of 1 for 24 h. **(B)** The effect of knockdown of gcARF1 on the expression of gcARF1 in CIK cells that were mock infected or infected with GCRV at an MOI of 1 for 24 h. **(C)** Crystal violet staining for knockdown of gcARF1 in CIK cells that were mock infected or infected with GCRV at an MOI of 1 for 24 h. **(D, E)** Virus yield for overexpression and knockdown of gcARF1 in CIK cells infected with GCRV at an MOI of 1 for 24 h. **(F, G)** IB analysis of VP3, VP5, NS80 and NS38 proteins regulated by overexpression of gcARF1 in CIK cells infected with GCRV. CIK cells seeded overnight in 6-well plates were transiently transfected with FLAG vector or gcARF1-FLAG (+: 500 ng, ++: 1000 ng). 24 h later, the cells were infected with the GCRV at an MOI of 1 or left untreated. The cells were collected at 12 **(F)** or 24 dpi **(G)** for protein extraction. **(H)** IB analysis of VP3, VP5, NS80 and NS38 proteins regulated by knockdown of gcARF1 in CIK cells infected with GCRV for 24 h. CIK cells seeded overnight in 6-well plates were transiently transfected with 100 nM control-siRNA or siARF1. After 24 h later, the cells were infected with the GCRV at an MOI of 1 or left untreated. The cells were collected at 24 hpi for protein extraction. **(I)** qRT-PCR analysis of *VP1*, *VP2*, *VP3*, *VP4*, *VP5*, *VP6*, *VP7*, *NS38* or *NS80* expression regulated by overexpression of gcARF1 in CIK cells infected with GCRV. CIK cells seeded overnight in 6-well plates were transiently transfected with FLAG vector or gcARF1-FLAG. After 24 h later, the cells were infected with the GCRV at an MOI of 1. Another 24 h later, these cells were collected and used for RNA extraction and qRT-PCR. **(J)** qRT-PCR analysis of *VP1*, *VP2*, *VP3*, *VP4*, *VP5*, *VP6*, *VP7*, *NS38* or *NS80* expression regulated by knockdown of gcARF1 in CIK cells infected with GCRV for 24 h. CIK cells seeded overnight in 6-well plates were transiently transfected with 100 nM control-siRNA or siARF1. After 24 h later, the cells were infected with the GCRV at an MOI of 1. Another 24 h later, these cells were collected and used for RNA extraction and qRT-PCR. Means ± SEM (n=3) are shown in **(B, D, E, I, J)**. Data were tested for statistical significance, **p < 0.01.

To explore the role of gcARF1 in GCRV infection, CIK cells were transfected with gcARF1-FLAG and then infected with GCRV. Compared with the control cells transfected with FLAG empty plasmid, severe cytopathic effect was observed after GCRV infection in the overexpression group (**Figure 1A**). Cells treated with gcARF1-specific siRNA had efficient reduction in expression of gcARF1 compared with control siRNA with or without GCRV infection (**Figure 1B**), and siRNA-mediated knockdown of gcARF1 expression showed much more resistant to GCRV infection than the cells transfected with control siRNA (**Figure 1C**). Consistent with these data, the overexpression of gcARF1 in CIK cells dramatically promoted the GCRV replication with the higher viral titers, and inhibited the GCRV replication by gcARF1-specific siRNA (**Figures 1D, E**).

Since that we have antibodies against VP3, VP5, NS80 and NS38 proteins of GCRV, the effects of gcARF1 on the protein expressions of 2 structural proteins and 2 nonstructural proteins of GCRV were examined. Antibody specificity was verified by immunoblotting in the mock-infected and GCRV-infected CIK cells. Using the anti-NS80 polyclonal rabbit antibody, anti-NS38 polyclonal mouse antibody, anti-VP3 polyclonal mouse antibody or anti-VP5 polyclonal rabbit antibody against GCRV-873 strain, a predicated size of approximately 80 kDa (**Figure S1A**), 40 kDa (**Figure S1B**), 130 kDa (**Figure S1C**) or 70 kDa (**Figure S1D**) was observed in the GCRV-infected CIK cells. The overexpression of gcARF1 increased the protein level of VP3, VP5, NS80 and NS38 in a dose dependent manner both at 12- and 24-hours post-infection (hpi) (**Figures 1F, G**). The knockdown of gcARF1 significantly decreased the protein level of these structural and nonstructural proteins of GCRV (**Figure 1H**).

To determine whether the overexpression or knockdown of gcARF1 had a similar effect at the mRNA level, the expression of 9 GCRV genes was examined by qRT-PCR. The overexpression of gcARF1 significantly increased the mRNA level of all tested genes including *VP1*, *VP2*, *VP3*, *VP4*, *VP5*, *VP6*, *VP7*, *NS38* and *NS80* for the transfected gcARF1 in a dose-dependent manner (**Figure 1I**), and the knockdown of gcARF1 inhibited significantly the expression of all these genes (**Figure 1J**). Taken together, these results clearly indicate that gcARF1 promotes virus replication and aggravates virus-induced cytopathogenicity in response to GCRV infection.

### NS80 and NS38 of GCRV recruit host GTPase gcARF1 to cytoplasmic VIBs through protein-protein interactions

**Figure 2:**
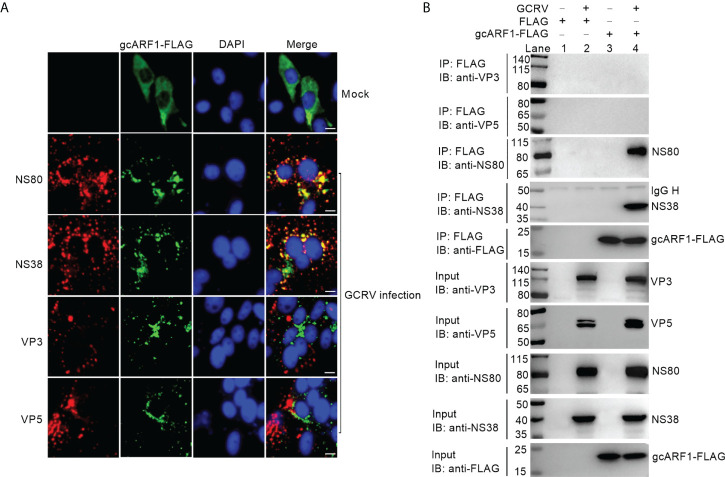
The subcellular co-localizations or interactions between gcARF1 and GCRV proteins. **(A)** The subcellular co-localizations between gcARF1 and GCRV proteins. CIK cells plated onto coverslips in 24-well plates were transfected with FLAG-tagged gcARF1. Then the cells were infected with the GCRV at an MOI of 1 or left untreated for another 24 h. Finally, the cells were washed and fixed with 4% PFA for immunofluorescence assays. The images were obtained by Leica confocal microscopy. Scale bars, 10 µm. **(B)** The interactions between gcARF1 and NS80, NS38, VP3 or VP5. CIK cells seeded in 10-cm^2^ dishes were transfected with indicated plasmids. After 24 h later, the cells were infected with the GCRV at an MOI of 1 or left untreated for another 24 h. Finally, the cells were harvested and used for protein extraction. Co-IP was performed with anti-FLAG-conjugated agarose beads. The cell lysates and bound proteins were analyzed by immunoblotting with the indicated Abs.

Previous works showed that NS80 and NS38 of GCRV could form cytoplasmic VIBs in the transfected or GCRV infected cells (13, 14, 20). We confirmed that ectopically expressed gcARF1 was diffusely distributed throughout the cytoplasm in the absence of infection, but formed a punctate distribution scattered throughout the cytoplasm in the case of GCRV infection (**Figure 2A**). The obvious colocalization between gcARF1 and NS80/NS38 was observed, but not for gcARF1 and VP3/VP5 (**Figure 2A**), which indicated that they were recruited to cytoplasmic VIBs.

To further confirm if gcARF1 was recruited by NS80 and NS38 of GCRV into cytoplasmic VIBs, we used the overexpressed NS80 or NS38 instead of GCRV infection to verify the effect of NS80 or NS38 on the localization of gcARF1. When the CIK cells were co-transfected with gcARF1-FLAG and NS80-GFP or NS38-GFP, gcARF1 was recruited into cytoplasmic VIBs by NS80 and NS38 of GCRV. The subcellular localizations of gcARF1 and NS80 completely overlapped in areas (**Figure S2A**).

To examine whether gcARF1 also bound to Golgi complex, we used Golgi complex marker to label the localization of Golgi complex. In the absence of infection, the Golgi complex was compact. In GCRV infected cells, Golgi complex became fragmented (**Figure S2B**), and a small amount of gcARF1 was localized at the Golgi complex (**Figure S2C**). Furthermore, we also observed that the staining of Golgi complex was not predominantly localized with cytoplasmic VIBs of GCRV (**Figure S2D**).

Previous studies showed that BFA could disrupt the structure of the Golgi apparatus (21, 22). We next investigated the effects of BFA treatment on the Golgi apparatus in the presence and absence of GCRV infection. The immunofluorescence results showed that both BFA treatment and GCRV infection caused the fragmentation of the Golgi complex. However, the BFA-induced fragmentation of the Golgi complex remained unchanged in the presence of GCRV infection (**Figure S2E**).

Next, we tested whether NS80 and NS38 of GCRV interacted with gcARF1. CIK cells were transfected with FLAG-tagged gcARF1, and then infected with GCRV or left untreated. The interactions between FLAG and NS80/NS38/VP3/VP5 were examined as the negative controls. As shown in **Figure 2B**, no NS80, NS38, VP3 or VP5 band was observed (Lane 2), which confirmed that GCRV proteins were not pull-down nonspecifically from the whole protein lysate. However, the anti-FLAG-M2 affinity gel-immunoprecipitated gcARF1 was associated with NS80 (Lane 4 using anti-NS80 antibody for IP product in **Figure 2B**) and NS38 (Lane 4 using anti-NS38 antibody for IP product in **Figure 2B**), but not with VP3 (Lane 4 using anti-VP3 antibody for IP product in **Figure 2B**) and VP5 (Lane 4 using anti-VP5 antibody for IP product in **Figure 2B**). Sequence analysis revealed that gcARF1 contained a small_GTP domain. A gcARF1 mutant, which only contained a small_GTP domain (**Figure S3A**), was sufficient for the associations with NS80 and NS38 of GCRV (**Figure S3B**).

Together, these data demonstrate that the nonstructural proteins NS80 and NS38 of GCRV recruit host gcARF1 to cytoplasmic VIBs through protein-protein interactions.

### gcARF1 activation by GBF1 promotes the generation of cytoplasmic VIBs during GCRV infection

**Figure 3:**
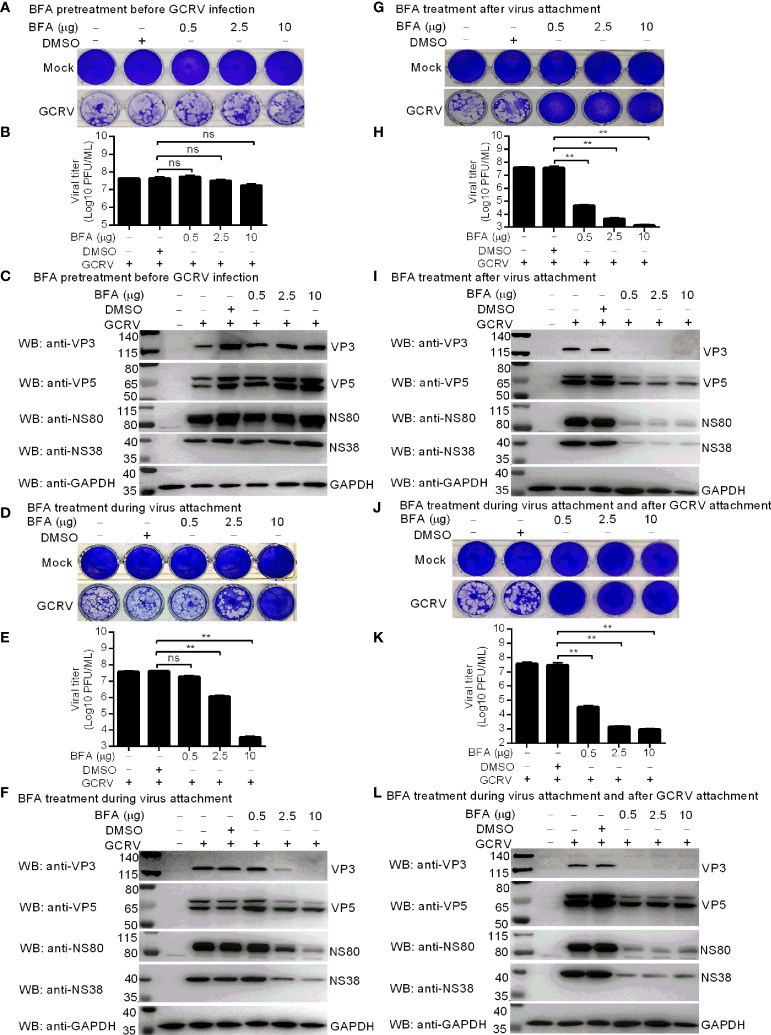
Inhibition of gcARF1 activation by BFA impairs GCRV replication and infection. **(A–C)** BFA pretreatment before GCRV infection has no influence on GCRV replication and infection. CIK cells plated in a 24-well or 6-well plates were incubated with BFA using the indicate concentrations or equivalent volume of DMSO for 1 h or left untreated. **(D-F)** BFA treatment during virus attachment suppressed GCRV replication and infection. CIK cells plated in a 24-well or 6-well plates were infected with GCRV and treated with BFA using the indicate concentrations or equivalent volume of DMSO for 1 h or left untreated. Then, the cells were washed with PBS to remove BFA and non-adsorbed virions. **(G–I)** BFA treatment after virus attachment suppressed GCRV replication and infection. CIK cells were infected with GCRV for 1 h, then washed with PBS to remove non-adsorbed virions, and finally treated with BFA using the indicate concentrations or equivalent volume of DMSO for another 24 h or left untreated. **(J–L)** BFA treatment during virus attachment and after virus attachment suppressed GCRV replication and infection. CIK cells were infected with GCRV and treated with BFA for 1 h, then washed with PBS to remove BFA and non-adsorbed virions, and finally treated with BFA for another 24 h. The cells in the 24-well plates were used for crystal violet staining **(A, D, G, J)**, the culture supernatants of infected cells used for determination of GCRV titers **(B, E, H, K)**, and the cells in the 6-well plates used for protein extraction **(C, F, I, L)**. Means ± SEM (n=3) are shown in **(B, E, H, K)**. Data were tested for statistical significance. The asterisk above the bracket indicated statistical significance between the two groups connected by the bracket. **p < 0.01; ns, not significant.

BFA inhibits ARF1 activation by targeting the guanine nucleotide exchange factor GBF1 (23, 24). To explore the role of gcARF1 activation in the viral replication cycle, CIK cells were treated with 0.5~10 μg/mL BFA, which had been confirmed to have no significant effect on the viability of CIK cells (**Figure S4A**). Treatment with BFA before GCRV infection had no obvious effect on the viral cytopathogenicity (**Figure 3A**), virus proliferation (**Figure 3B**) and the expressions of VP3, VP5, NS80 and NS38 (**Figure 3C**). Treatment with BFA during viral attachment inhibited the replication and infection of GCRV in a dose dependent manner. Low concentration of BFA had no obvious effect on the viral cytopathogenicity, virus proliferation and the expressions of viral proteins. However as the concentration of BFA increased, the inhibition of BFA on the viral replication and infection was more obvious (**Figures 3D–F**). Treatment with BFA after viral attachment significantly led to the inhibition of GCRV replication and infection (**Figures 3G–L**). These results indicate that gcARF1 activation by GBF1 promotes GCRV replication and infection through facilitating the entry and proliferation of GCRV lifecycle.

**Figure 4:**
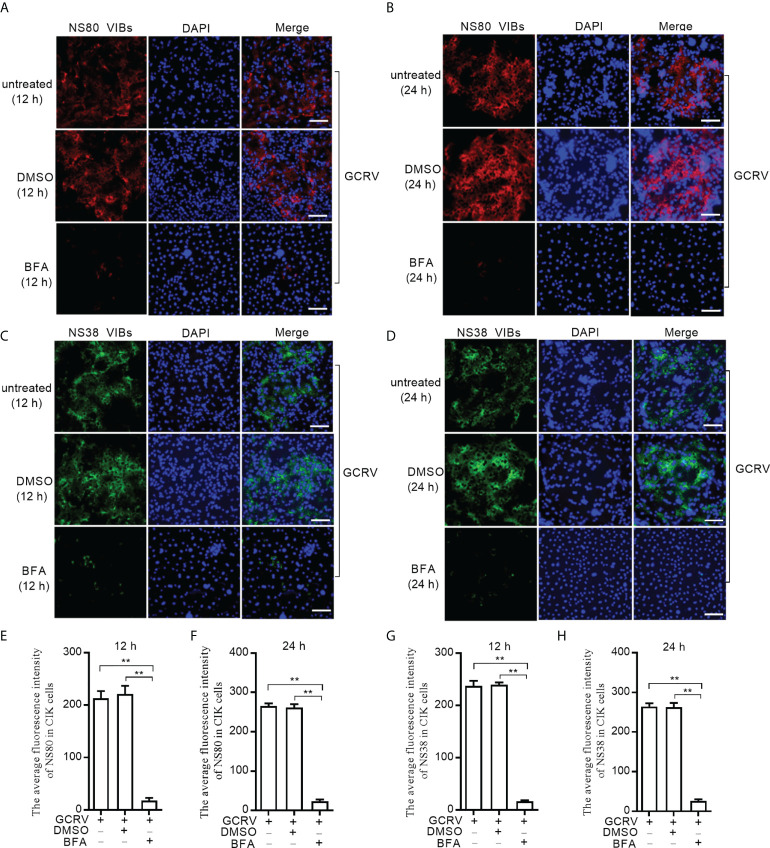
BFA treatment reduces the numbers of viral inclusion bodies. **(A)** Immunofluorescence analysis for NS80 in CIK cells that were treated with DMSO or BFA for 12 h or left untreated. Scale bars, 50 µm. **(B)** Immunofluorescence analysis for NS80 in CIK cells that were treated with DMSO or BFA for 24 h or left untreated. Scale bars, 50 µm. **(C)** Immunofluorescence analysis for NS38 in CIK cells that were treated with DMSO or BFA for 12 h or left untreated. Scale bars, 50 µm. **(D)** Immunofluorescence analysis for NS38 in CIK cells that were treated with DMSO or BFA for 24 h or left untreated. Scale bars, 50 µm. **(E)** The average fluorescence intensity of NS80 in CIK cells that were treated with DMSO or BFA for 12 h or left untreated. **(F)** The average fluorescence intensity of NS80 in CIK cells that were treated with DMSO or BFA for 24 h or left untreated. **(G)** The average fluorescence intensity of NS38 in CIK cells that were treated with DMSO or BFA for 12 h or left untreated. **(H)** The average fluorescence intensity of NS38 in CIK cells that were treated with DMSO or BFA for 24 h or left untreated. Means ± SEM (n=3) are shown in **(E-H)**. Data were tested for statistical significance. The asterisk above the bracket indicated statistical significance between the two groups connected by the bracket. **p < 0.01.

Since NS80 and NS38 of GCRV recruit gcARF1 to VIBs by protein-protein interactions, we further investigated the effect of host gcARF1 in the formation or generation of cytoplasmic VIBs in GCRV-infected cells. CIK cell infected by GCRV for 1 h were treated with BFA or DMSO or left untreated. These cells were subsequently fixed and processed for immunofluorescence using antibodies both against NS80 and NS38 of GCRV, which served as protein markers for VIBs of GCRV. Treatment of cells with BFA for 12 or 24 h led to the expected decrease or disappearance of VIBs compared with the untreated cells or DMSO-treated cells (**Figure 4**). Previous studies have revealed that the outer-capsid proteins of reovirus are responsible for initiating infection. VP5 is the outer-capsid protein of GCRV, and autocleavage of VP5 has been confirmed to be critical for aquareovirus to initiate efficient infection (25). BFA treatment also significantly inhibited the numbers of fluorescent cells expressed with VP5 (**Figures S4B–D**).

### Crystal structures of gcARF1 and gcARF1-GDP complex

The data processed by auto-PROC_XDS is used. The space group is C 1 2 1, each asymmetric unit of the gcARF1 crystal contains two copies of molecules, and the solvent content is 42%. Auto-build and refinement programs from Phenix software were used to reconstruct the structure of gcARF1, with the R-free value of 0.2457 and R-work value of 0.2029. The gcARF1 protein contained a seven-stranded β-sheet surrounded by six α-helices (**Figure S5**), which indicated that the overall structure of gcARF1 was similar to those of other ARF1 proteins.

**Figure 5:**
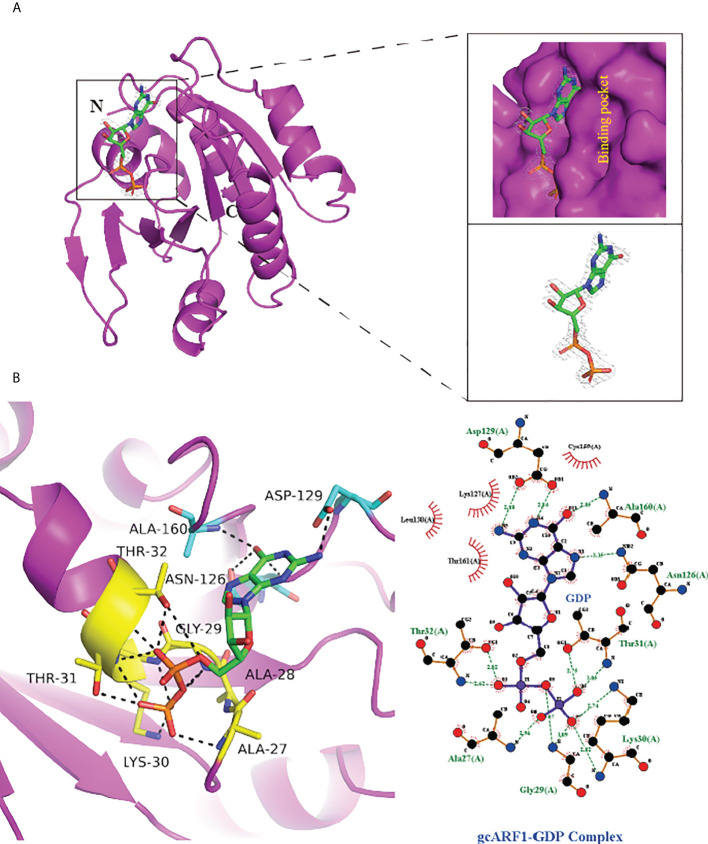
Structural analysis of gcARF1-GDP complex. **(A)** The GDP combinative pocket of gcARF1. The surface diagram is shown in the upper right, and the GDP electron density shown in the lower right (level = 2.0). **(B)** The hydrogen bond interaction between GDP and gcARF1. Left: The GDP is shown as green sticks, the AAGKTT motif shown as yellow sticks, the N^126^, D^129^ and A^160^ shown as cyan sticks, and the hydrogen bonds shown as black dotted line. Right: The interaction between gcARF1 and GDP analyzed by LigPlus software.

During the expression of gcARF1 protein in *E. coli*, we found that the A260 absorbance of gcARF1 protein was unusually high (A260/A280 = 0.82, which was about 0.5 for general proteins), suggesting that gcARF1 might bind to nucleotide or nucleotide analogue when expressed in the *E. coli* system. Meanwhile, the crystal structure of gcARF1 has redundant electron density. After repeated refined calculation of the gcARF1 structure, it was confirmed that the excess electron cloud density could match GDP perfectly. The structure of gcARF1-GDP complex was finally confirmed (**Figure 5**). The structural analysis showed that the groove of gcARF1 binding to GDP mainly consisted of loop between β1 and α2, partial α2, loop between β6 and α5, and loop between β7 and α6. The interaction between gcARF1 and GDP was further analyzed by LigPlus software. Eight amino acid residues (A^27^, G^29^, K^30^, T^31^, T^32^, N^126^, D^129^ and A^160^) were involved in the binding of GDP with gcARF1. The N^126^, D^129^ and A^160^ of gcARF1 interacted with guanosine group of GDP by hydrogen bond, which included the carbonyl group of N^126^ side chain interacting with the carbonyl group of GDP guanosine group, the hydrogen atom of the amino group of N^126^ side chain interacting with the nitrogen atom of GDP guanosine group, the oxygen atom of the carbonyl group of D^129^ side chain interacting with the amino hydrogen atom of GDP guanosine group, and the amino group of A^160^ main chain interacting with the carbonyl group of GDP guanosine group. Importantly in the ^27^AAGKTT^32^ motif, the oxygen atom of the T^32^ side chain interacting with the oxygen atom of the first phosphate group of GDP, the hydrogen atom of the K^30^ side chain amino group and the oxygen atom of the T^31^ side chain interacting with the oxygen atom of the second phosphate group of GDP were observed through hydrogen bonds. Therefore, the ^27^AAGKTT^32^ motif may be crucial for the gcARF1 binding to GDP and the function of gcARF1.

**Figure 6:**
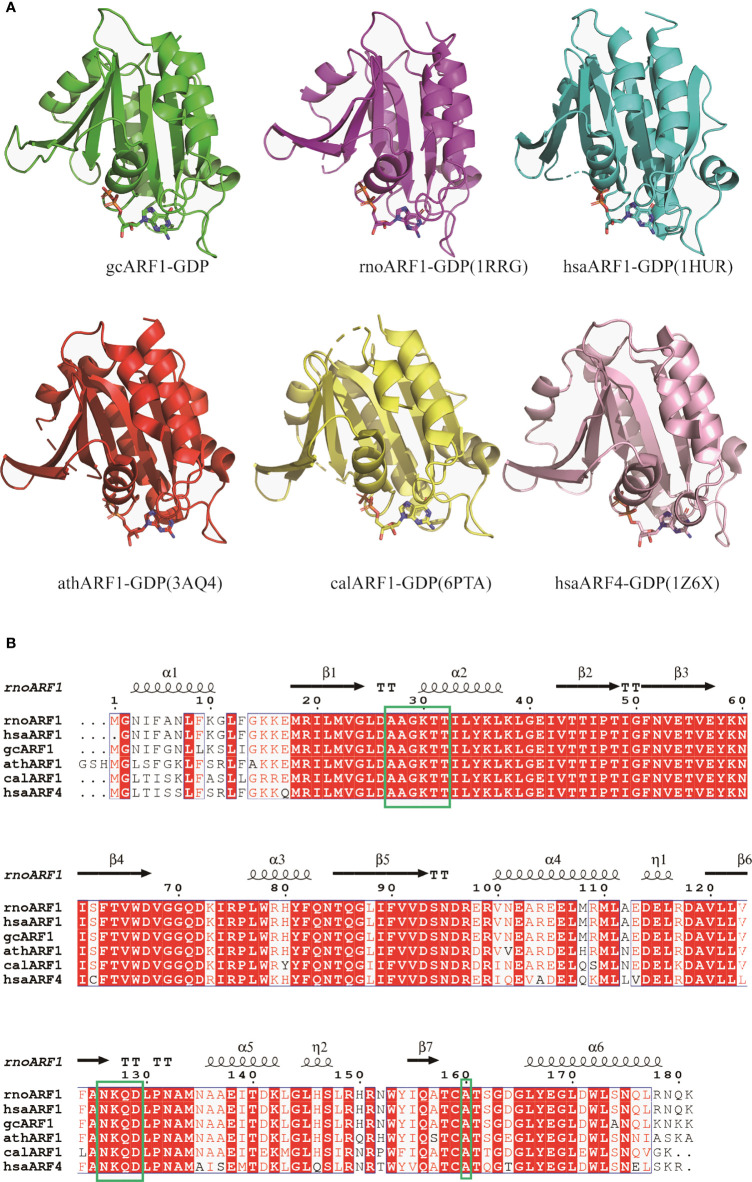
Structure and sequence alignments of gcARF1-GDP and ARF-GDP from other species. **(A)** Structure alignments of gcARF1-GDP (green), *Rattus norvegicus* ARF1-GDP (PDB code: 1RRG, purple, RMSD = 0.5), *Homo sapiens* ARF1-GDP (PDB code: 1HUR, cyan, RMSD = 0.6), *Arabidopsis thaliana* ARF1-GDP (PDB code 3AQ4, red, RMSD = 0.8), *Candida albicans* SC5314 ARF1-GDP (PDB code: 6PTA, yellow, RMSD = 0.7) and *Homo sapiens* ARF4-GDP (PDB code: 1Z6X, pink, RMSD = 1.1). **(B)** Sequence alignments of gcARF1 and ARF proteins from other species by Clustal W and ESPript 3.0.

To further investigate whether the AAGKTT motif of ARF1 binding to GDP are conversed among different species, structural comparisons were analyzed using DALI server. The top 5 most similar to gcARF1 structure are *Rattus norvegicus* ARF1 (PDB code: 1RRG), *Homo sapiens* ARF1 (PDB code: 1HUR), *Arabidopsis thaliana* ARF1 (PDB code 3AQ4), *Candida albicans* SC5314 ARF1 (PDB code: 6PTA) and *Homo sapiens* ARF4 (PDB code: 1Z6X) (**Figure 6A**). Structure alignment analysis suggested that the binding sites between ARF1 and GDP are similar in different species. The AAGKTT motif locating at between TT loop and α2 is also conserved among different species (**Figure 6B**). All these results suggest that the mechanism of gcARF1 binding to GDP is evolutionarily conservative and the ^27^AAGKTT^32^ motif is essential for gcARF1 binding to GDP.

### The ^27^AAGKTT^32^ motif and T^31^ residue are required for the function of gcARF1 in promoting GCRV replication and infection

**Figure 7:**
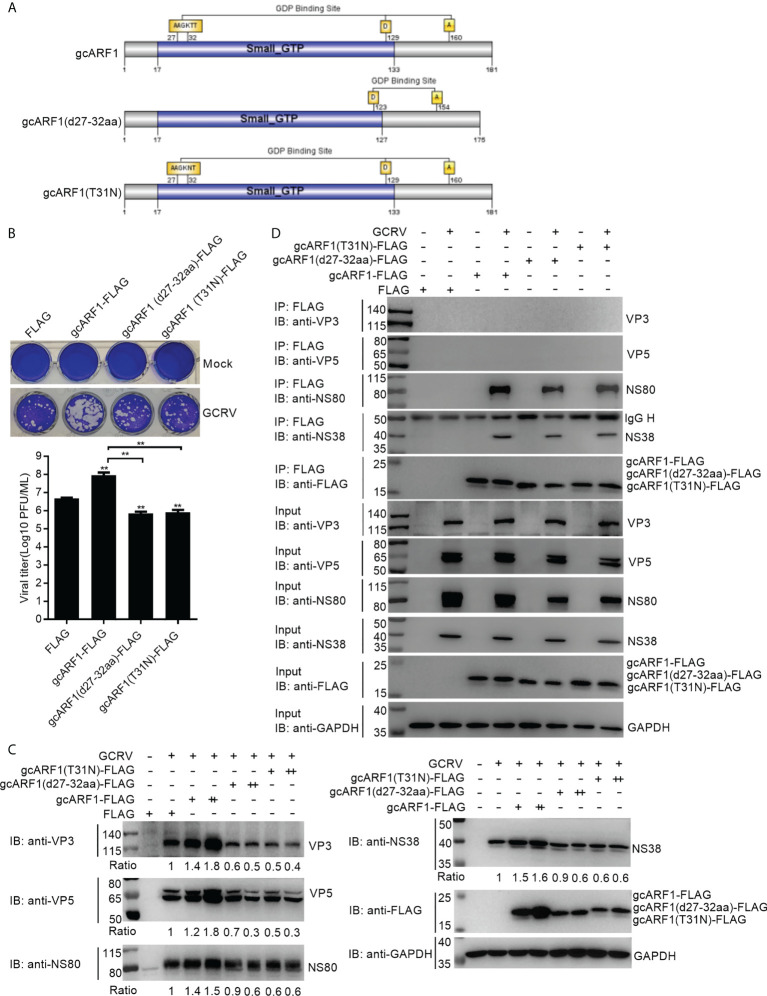
The ^27^AAGKTT^32^ motif and T^31^ residue are required for the function of gcARF1 in promoting GCRV replication and infection. **(A)** Schematic representation of the gcARF1 and its mutants. **(B)** Crystal violet staining and determination of GCRV titers for overexpression of gcARF1 mutants including gcARF1(d27-32aa) and gcARF1(T31N) in CIK cells that were infected with GCRV at an MOI of 1 for 24 h. The asterisk above the error bars indicated statistical significance using the group transfected with FLAG as the control group. The asterisk above the bracket indicated statistical significance between the two groups connected by the bracket. **(C)** IB analysis of VP3, VP5, NS80 and NS38 proteins regulated by overexpression of gcARF1 or gcARF1 mutants including gcARF1(d27-32aa) and gcARF1(T31N) in CIK cells infected with GCRV. CIK cells seeded overnight in 12-well or 6-well plates were transiently transfected with indicated plasmids. After 24 h later, the cells were infected with the GCRV at an MOI of 1 or left untreated. The supernatants in 12-well plates were collected at 24 hpi for viral titer assays, and the cells were fixed and stained with crystal violet **(B)**. The cells in 6-well plates were collected at 24 hpi for protein extraction **(C)**. +: 500 ng, ++: 1000 ng. The expression ratios for viral proteins were quantified by Quantity One. **(D)** The interactions between gcARF1, gcARF1(d27-32aa) or gcARF1(T31N) and viral proteins. CIK cells seeded in 10-cm^2^ dishes were transfected with the indicated plasmids. After 24 h later, the cells were infected with or without the GCRV at an MOI of 1. Then the cells were harvested and lysed at 24 hpi. Co-IP was performed with anti-FLAG-conjugated agarose beads. The cell lysates and bound proteins were analyzed by immunoblotting with the indicated Abs.

In mammals, ARF1(T31N), a mutant that preferentially binds GDP, is the activation-impaired form of ARF1 (26). Here, crystal structure of gcARF1-GDP complex revealed that the ^27^AAGKTT^32^ motif was essential for gcARF1 binding to GDP. To determine the pivotal domain, motif and/or residue(s) affecting the function of gcARF1, three mutants included gcARF1-small_GTP-FLAG (**Figure S3A**), gcARF1(d27-32aa)-FLAG and gcARF1(T31N)-FLAG (**Figure 7A**) were constructed. We firstly investigated the role of small_GTP domain of gcARF1 in GCRV replication and infection. Similar to gcARF1, overexpression of small_GTP domain of gcARF1 increased the cytopathic effect caused by GCRV infection, with the higher viral titers compared with the control cells transfected with FLAG empty plasmid (**Figures S6A, S6B**). Overexpression of small_GTP domain of gcARF1 also promoted the expressions of virus-related proteins (**Figure S6C**). However, the deletion of ^27^AAGKTT^32^ motif of gcARF1 and the mutation of ARF1(T31N) significantly inhibited GCRV replication (**Figure 7B**). Furthermore, the deletion of ^27^AAGKTT^32^ motif of gcARF1 or the mutation of ARF1(T31N) also inhibited the expressions of virus-related proteins (**Figure 7C**), which were opposite for the roles of gcARF1 or small_GTP domain of gcARF1 in GCRV infection (**Figures 1D-G**, **Figure S6**). All these data suggest that the small_GTP domain of gcARF1 is crucial for GCRV replication and infection, and that the ^27^AAGKTT^32^ motif and the amino acid residue T31 of gcARF1 are indispensable for the function of gcARF1 in promoting GCRV replication and infection.

Since the above results from Co-IP assays revealed that the small_GTP domain of gcARF1 was sufficient for the association between gcARF1 and NS80 or NS38 protein of GCRV, we further investigated whether the ^27^AAGKTT^32^ motif and the amino acid residue T^31^ of gcARF1 were essential for protein-protein interactions between gcARF1 and NS80 or NS38 protein of GCRV. We found that the deletion of ^27^AAGKTT^32^ motif of gcARF1 or the mutation of ARF1(T31N) did not lead to the loss of the interaction with NS80 and NS38 proteins of GCRV (**Figure 7D**).

### The ^27^AAGKTT^32^ motif and T^31^ residue are required for the generation of VIBs

**Figure 8:**
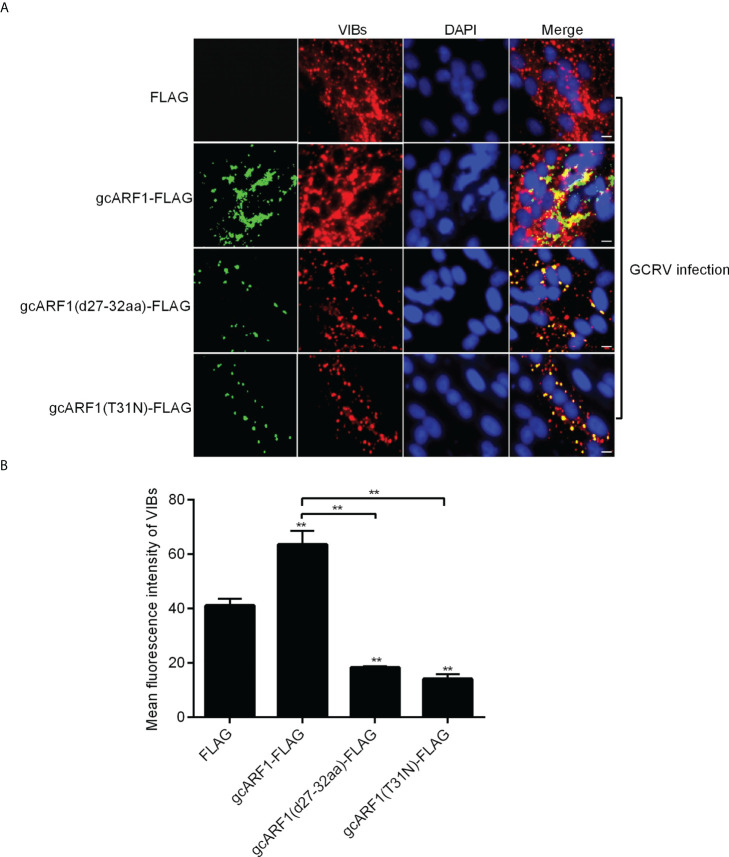
The ^27^AAGKTT^32^ motif and T^31^ residue are required for the generation of VIBs. **(A)** Immunofluorescence analysis for VIBs in CIK cells that were transfected with FLAG, gcARF1-FLAG, gcARF1(d27-32aa)-FLAG or gcARF1(T31N)-FLAG, respectively. Scale bars, 10 µm. **(B)** The average fluorescence intensity of VIBs in CIK cells that were transfected with FLAG, gcARF1-FLAG, gcARF1(d27-32aa)-FLAG or gcARF1(T31N)-FLAG, respectively. Data were tested for statistical significance. **p < 0.01. The asterisk above the error bars indicated statistical significance using the group transfected with FLAG as the control group. The asterisk above the bracket indicated statistical significance between the two groups connected by the bracket.

Given the role of gcARF1 in promoting the generations of cytoplasmic VIBs, we further investigated whether the ^27^AAGKTT^32^ motif and the amino acid residue T^31^ of gcARF1 were required for the formation and generation of VIBs. In consistent with the fact that the deletion of ^27^AAGKTT^32^ motif of gcARF1 or the mutation of ARF1(T31N) did not lead to the loss of the interaction with NS80 and NS38 proteins of GCRV, the localization of gcARF1 in cytoplasmic VIBs remained unaffected by the depletion of ^27^AAGKTT^32^ motif or the mutation of T^31^ residue (**Figure 8A**). However the total amounts of VIBs were significantly decreased by the depletion of ^27^AAGKTT^32^ motif or the mutation of T^31^ residue (**Figure 8B**). All these results suggest that the ^27^AAGKTT^32^ motif and T^31^ residue are required for the generation of VIBs.

## Discussion

During infection, many viruses replicate in cytoplasm of host cells and form viroplasms, viral factories or VIBs, which are often composed of membranous scaffolds, viral and cellular factors. VIBs have multiple functions, including the recruitment of viral and host factors to ensure efficient replication and assembly of virus particles and sequestration of viral nucleic acids and proteins from host innate immune responses (27–29). Previous studies have shown that NS80 of GCRV can form VIBs, and recruit all the inner-capsid proteins (VP1-VP4 and VP6) and NS38 into VIBs (13, 14). Our recent report revealed that NS80 and NS38 of GCRV can hijack grass carp TBK1 and IRF3 into cytoplasmic VIBs for decreasing the formation of TBK1-containing functional complexes and preventing IRF3 translocation into the nucleus, which ultimately leads to the impaired interferon antiviral response (30). Here, we firstly demonstrate that GCRV uses NS80 and NS38 to recruit host GTPase gcARF1 into VIBs to promote GCRV replication and infection. Crystallographic data and functional analysis reveal the pivotal role of ^27^AAGKTT^32^ motif and T^31^ residue of gcARF1 in the binding to GDP and GCRV replication and infection.

The GCRV genome encodes several non-structural proteins, which do not constitute the nucleocapsids of the virus, but are indispensable for the replication, proliferation, invasion and immune escape of GCRV. NS38 is one of non-structural proteins encoded by GCRV. It has been reported that NS38 interacts with inner-capsid proteins and NS80-RNA complex, and knockdown of NS38 can significantly inhibit the proliferation of GCRV (16). It is speculated that the effects of NS38 on viral protein synthesis are due to its RNA binding characteristics for facilitating interactions with host translational factors such as eIF3A, which is essential for viral translation initiation (16, 31). NS80 is the largest non-structural protein of GCRV. The N-terminal domain of NS80 can recruit NS38, VP1, VP2, VP4 and VP6 into VIBs, and its C-terminal domain is responsible for the formations of VIBs (13, 15, 32, 33). The ARF family is one of five subfamilies of Ras GTPase superfamily, which can cycle between an active GTP-bound state and an inactive GDP-bound state. Previous studies have shown that ARF1 protein can be localized to the Golgi complex, and regulates phosphatidylinositol 4-kinase IIIbeta activity, Golgi transport complex recruitment, architecture of ER-Golgi intermediate compartment, and the formation of bidirectional tubules from Golgi (7, 34, 35). In addition, it has been reported that ARF1 is also involved in the replication process of many viruses, including Hepatitis C virus (HCV), enterovirus 71, white spot syndrome virus (WSSV), and red clover necrotic mosaic virus (RCNMV) (9, 36–38). ARF proteins are also recruited into replication organelles or regulate membrane traffic between ER, ERGIC and Golgi to generate compartments for the replication of viruses (3, 39, 40). In this study, we firstly confirmed that the piscine ARF1 was recruited by NS80 and NS38 of GCRV into cytoplasmic VIBs *via* protein-protein interactions, and promoted GCRV replication and infection through facilitating the entry and proliferation processes of GCRV lifecycle.

Structure and sequence comparison showed that gcARF1 had high homology with lower eukaryotes (yeast), plants (Arabidopsis), mammals (mouse and human) and other species. GTP-binding domain contains three consensus elements GXXXXGK (S/T), DXXG and NKXD (41). The GXXXXGK (S/T) (where X is any residue) motif is known as a Walker A motif, which is also referred to as ‘phosphate-binding loop’ and thought to bind to the phosphate groups of GTP (42, 43). The NKXD (where X is any residue) motif can interact with the guanine ring (44). The GXXXXGK (S/T) and NKXD motifs are very conserved for ARF1 proteins from different species, with the same GLDAAGKT sequences for GXXXXGK (S/T) motif and NKQD for NKXD motif. Among eight amino acid residues (A^27^, G^29^, K^30^, T^31^, T^32^, N^126^, D^129^ and A^160^) involved in the binding of GDP with gcARF1, six amino acid residues locate within the two motifs. Therefore similar to mammal homologues, gcARF1 may act as a molecular switch by switching between an active GTP-bound state and an inactive GDP-bound state and may have undergone conformational changes to change its affinity for substrates through its conserved structural motifs. Furthermore, since the ^27^AAGKTT^32^ motif and T^31^ residue are essential for gcARF1 binding to GDP, the inhibition on the GCRV replication caused by the deletion of ^27^AAGKTT^32^ motif of gcARF1 and the mutation of ARF1(T31N) suggest that the GTPase activity of gcARF1 is important for GCRV replication and infection. However, the deletion of ^27^AAGKTT^32^ motif of gcARF1 and the mutation of ARF1(T31N) did not impair the interaction between gcARF1 and NS80/NS38 protein of GCRV. Based on these data, it is interesting to further resolve the crystal structure of gcARF1-NS80 or gcARF1-NS38 complex and compare the conformational differences between gcARF1 bound to viral protein and bound to GDP, which are helpful for revealing the molecular mechanism by which NS80 and NS38 proteins of GCRV recruit gcARF1 and promote the generation of VIBs.

The GTPase activity of ARF family is regulated by GEFs and GAPs, and lots of inhibitors targeting ARF, ARF-GEF complex, GEFs and GAPs have been reported (1). NAV-2729, which can bind to human ARF6 in the GEF binding region and thus inhibit the interaction of ARF6-GEF, has been used in the treatment of uveal melanoma (1, 45). The most commonly used inhibitor for ARF-GEF binding is BFA, a fungal macrolide that can be embedded in the hydrophobic groove at the binding interface between GEFs (Sec7) and ARF1, thereby inhibiting the GTP/GDP exchange of ARF1 (46). Although the replications of several viruses such as turnip mosaic virus (TuMV), coxsackievirus B3 (CVB3) and EMCV have been shown to be insensitive to BFA (47, 48), BFA treatment has been widely used to inhibit viral replication process in mammals. For example, the enteroviral protein 3A specifically triggers the recruitment of GBF1 to membranes to promote the replication of viral RNA; however BFA can block enterovirus replication by inhibiting the activity of GBF1 (47). For rotavirus, BFA could impair the yield of viral progeny *via* interfering with the synthesis of GBF1 and the virus assembly process (49, 50). During infectious bursal disease virus (IBDV) infection, interfering with GBF1 activity by BFA treatment leads to a dramatic change in the location of viral replication complexes, and significantly reduces the yield of infectious viral progeny (51). The present study revealed that inhibition of gcARF1 activity using BFA disrupted the generation of cytoplasmic VIBs in GCRV-infected cells, and alleviated the replication and infection of GCRV. Furthermore, the mechanisms controlling the GTPase activity of ARF1 may be very conserved, which are revealed by structure and sequence comparison of ARF1 proteins from grass carp and other species. It is interesting to further know whether many other inhibitors targeting ARF-GEF interaction such as AMF-26 can be used for prevention and treatment of grass carp hemorrhagic disease (52).

Several studies have indicated that ARF1 is critical for maintaining Golgi structure and function. The primary localization of mammalian ARF1 in cells is at the Golgi. During its GTP cycle, ARF1 reversibly associates with Golgi membranes, with the ARF1-GTP bound to the membrane and ARF1-GDP being cytosolic (53). Intriguingly, Golgi fragmentation and rearrangement have been observed during viral infections (54, 55). In response to the severe acute respiratory syndrome-associated coronavirus (SARS-CoV) infection, overexpression of ARF1 can restore Golgi morphology (56). Furthermore, many positive-sense RNA (+RNA) viruses form the replication complexes (RCs) for their replication, but ARF1 was hardly recruited to coronavirus RCs (57, 58). Similar to coronavirus mouse hepatitis virus (MHV), GCRV infection also caused Golgi fragmentation and rearrangement. However, gcARF1 was recruited into cytoplasmic VIBs by viral proteins, which was different from the previous report (57). Our results, together with those of others, reveal that ARF1 utilizes distinct means to target different endomembrane recruitment for conferring advantages for viral replication and infection.

In summary, here we demonstrate that gcARF1, which is recruited to cytoplasmic VIBs by NS80 and NS38 of GCRV, promotes GCRV replication and infection through facilitating the entry and proliferation processes of GCRV lifecycle. The AAGKTT motif and the amino acid residue T^31^ located in the small_GTP domain of gcARF1 are indispensable for the function of gcARF1 in viral replication and infection. Further investigations are needed to unravel whether other ARF proteins contribute to the biogenesis of functional VIBs and to GCRV infection.

## Data availability statement

The datasets presented in this study can be found in online repositories. The names of the repository/repositories and accession number(s) can be found in the article/**Supplementary Material**.

## Author contributions

JZ, PL, and MC designed the study. JZ performed most of the experiments. PL and RL performed some of the experiments. SO and MC provided reagents or assistance. JZ, PL, and MC analyzed the data and prepared the Figures. JZ, PL, and MC wrote the manuscript. All authors contributed to the article and approved the submitted version.

## Funding

This work was supported by Strategic Priority Research Program of the Chinese Academy of Sciences Grant XDA24010308, the National Key Research and Development Program of China (Grant Nos. 2019YFD0900703 and 2021YFC2301403), the National Natural Science Foundation of China (Grant Nos. 82225028 and 82172287), and Fujian Science and Technology Department Project (Grant No. 2020Y4007).

## Conflict of interest

The authors declare that the research was conducted in the absence of any commercial or financial relationships that could be construed as a potential conflict of interest.

## Publisher’s note

All claims expressed in this article are solely those of the authors and do not necessarily represent those of their affiliated organizations, or those of the publisher, the editors and the reviewers. Any product that may be evaluated in this article, or claim that may be made by its manufacturer, is not guaranteed or endorsed by the publisher.
