# Supplementary material for: Structural and functional analysis of the small GTPase ARF1 reveals a pivotal role of its GTP-binding domain in controlling of the generation of viral inclusion bodies and replication of grass carp reovirus
Source: Front Immunol. 2022 Aug 26;13:956587. doi: 10.3389/fimmu.2022.956587 (PMC9459132; doi:10.3389/fimmu.2022.956587)
Supplement: Supplementary file 1 [file DataSheet_1.zip › Supplementary Materials-20220802.docx]

**Figure S1. The validation of antibody specificity.**

(A) The validation of anti-NS80 polyclonal rabbit antibody in CIK cells with or without GCRV infection. (B) The validation of anti-NS38 polyclonal mouse antibody in CIK cells with or without GCRV infection. (C) The validation of anti-VP3 polyclonal mouse antibody in CIK cells with or without GCRV infection. (D) The validation of anti-VP5 polyclonal rabbit antibody in CIK cells with or without GCRV infection.

**Figure S2. The localizations of gcARF1 and NS80 or NS38 protein of GCRV.**

1. The subcellular co-localizations of gcARF1 and NS80 or NS38 protein of GCRV. (B) The effect of GCRV infection on the subcellular distribution of Golgi complex. (C) The subcellular co-localizations of gcARF1 and Golgi complex. (D) The subcellular co-localizations of NS80 and Golgi complex. (E) The effect of BFA on the subcellular distribution of Golgi complex in the presence and absence of GCRV infection. Scale bars, 10 µm.

**Figure S3. The small_GTP domain of gcARF1 is sufficient for the interactions with NS80 and NS38 of GCRV.**

(A) Schematic representation of the gcARF1 and its mutant. (B) The interactions between gcARF1 or the small_GTP domain of gcARF1 and viral proteins.

**Figure S4. BFA treatment inhibits the numbers of fluorescent cells expressed with VP5.**

1. The effect of BFA treatment on the cell viability. (B) Immunofluorescence analysis for VP5 in CIK cells that were treated with DMSO or BFA for 12 h or left untreated. Scale bars, 50 µm. (C) Immunofluorescence analysis for VP5 in CIK cells that were treated with DMSO or BFA for 24 h or left untreated. Scale bars, 50 µm. (D) The average fluorescence intensity of VP5 in CIK cells that were treated with DMSO or BFA for 24 h or left untreated.

**Figure S5. Crystal structure of gcARF1.**

(A) Crystal structure and secondary structure distribution of gcARF1 protein. (B) Schematic diagram of gcARF1 secondary structure distribution. Purple cylinders represent alpha helices and orange sheets represent beta folds.

**Figure S6. The effect of small_GTP domain of gcARF1 in GCRV replication and infection.**

(A) Crystal violet staining for overexpression of gcARF1 or small_GTP domain of gcARF1 in CIK cells that were mock infected or infected with GCRV at an MOI of 1 for 24 h. (B) Virus yield for overexpression of gcARF1 or small_GTP domain of gcARF1 in CIK cells infected with GCRV at an MOI of 1 for 24 h. (C) IB analysis of VP3, VP5, NS80 and NS38 proteins regulated by overexpression of gcARF1 or small_GTP domain of gcARF1 in CIK cells infected with GCRV. +: 500 ng, ++: 1000 ng.
