# Supplementary material for: Structural and functional analysis of the small GTPase ARF1 reveals a pivotal role of its GTP-binding domain in controlling of the generation of viral inclusion bodies and replication of grass carp reovirus
Source: Front Immunol. 2022 Aug 26;13:956587. doi: 10.3389/fimmu.2022.956587 (PMC9459132; doi:10.3389/fimmu.2022.956587)
Supplement: Supplementary file 1 [file DataSheet_1.zip › Table S1.docx]

**Table S1. Primers used for the present study.**

| Name | Sequence | Application |
| --- | --- | --- |
| gcARF1F | CGGAATTCATGGGGAACATTTTTGGC | Ligated to p3xFLAG-CMV™-14 vector |
| gcARF1R | CGGGATCCTTTCTTGTTCTTGAGCTG |  |
| gcARF1-small_GTPF | CGGAATTCATGGAGATGCGGATTTTG |  |
| gcARF1-small_GTPR | CGGGATCCTGCATTTGGCAGATCCTG |  |
| gcARF1(d27-32aa)F | ATGGTTGGGCTGGATATCCTGTACAAACTG |  |
| gcARF1(d27-32aa)R | CAGTTTGTACAGGATATCCAGCCCAACCATC |  |
| gcARF1(T31N)F | AGCTGGGAAAAACACAATCCTGTAC |  |
| gcARF1(T31N)R | TACAGGATTGTGTTTTTCCCAGCTG |  |
| gcARF1F1 | CGGAATTCATGGGGAACATTTTTGGC | Ligated to pTurboGFP-N vector |
| gcARF1R1 | CGGGATCCAATTTCTTGTTCTTGAGCTG |  |
| gcARF1F2 | CGCGGATCCATGGGGAACATTTTTGGCAACC | Ligated to pET28a-SUMO vector |
| gcARF1R2 | CCGCTCGAGTCATTTCTTGTTCTTGAGCTGG |  |
| VP1F | TACCAACCCGTTAGTGCTT | Quantitative real-time PCR |
| VP1R | GGAGTAGTAGAATACCGTGGC |  |
| VP2F | TACGCCTACACCTTACTTCAA |  |
| VP2R | CGGTTCGGTCCACTCTATT |  |
| VP3F | GCTTTCTTCATCCGAGTGG |  |
| VP3R | GCGACGAGGACATTGGTA |  |
| VP4F | TGGCTCTATTGATGTCTGATG |  |
| VP4R | CAGTGATGTGGACGAAAGG |  |
| VP5F | CGCCATCAATCTCGCTATCA |  |
| VP5R | CGCCCTTGTATGTCGTCTCA |  |
| VP6F | CCCTGACTGGACGCCTAA |  |
| VP6R | CGCCTGCCACTTCTACGA |  |
| VP7F | AGCCATTCGCTCATTAGTCG |  |
| VP7R | TTTGGTGGGATGCTCGTTAG |  |
| NS38F | CTATGGCACTGGCGTTTA |  |
| NS38R | GTCGGGTAGTTCAGAGGG |  |
| NS80F | GGAAGCCGACAAGGGAATG |  |
| NS80R | TGGAGTAGCCGTGGGAAG |  |
| 18S rRNAF | ATTTCCGACACGGAGAGG |  |
| 18S rRNAR | CATGGGTTTAGGATACGCTC |  |
| EF1αF | CAGCACAAACATGGGCTGGTTC |  |
| EF1αR | ACGGGTACAGTTCCAATACCTCCA |  |
| β-ActinF | CACTGTGCCCATCTACGAG |  |
| β-ActinR | CCATCTCCTGCTCGAAGTC |  |
