# Supplementary figures and images for: Structural and functional analysis of the small GTPase ARF1 reveals a pivotal role of its GTP-binding domain in controlling of the generation of viral inclusion bodies and replication of grass carp reovirus
Source: Front Immunol. 2022 Aug 26;13:956587. doi: 10.3389/fimmu.2022.956587 (PMC9459132; doi:10.3389/fimmu.2022.956587)

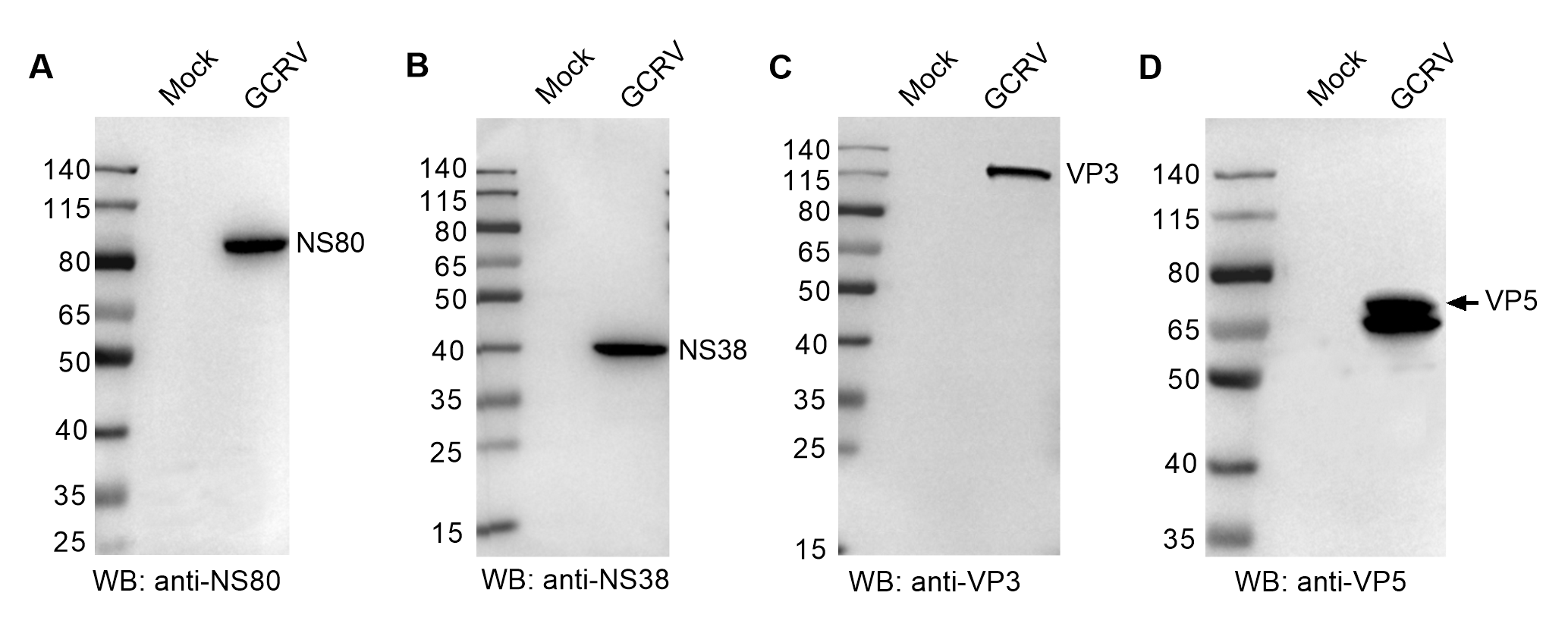

Supplement: Supplementary file 1 [file DataSheet_1.zip › Figure S1.tif]

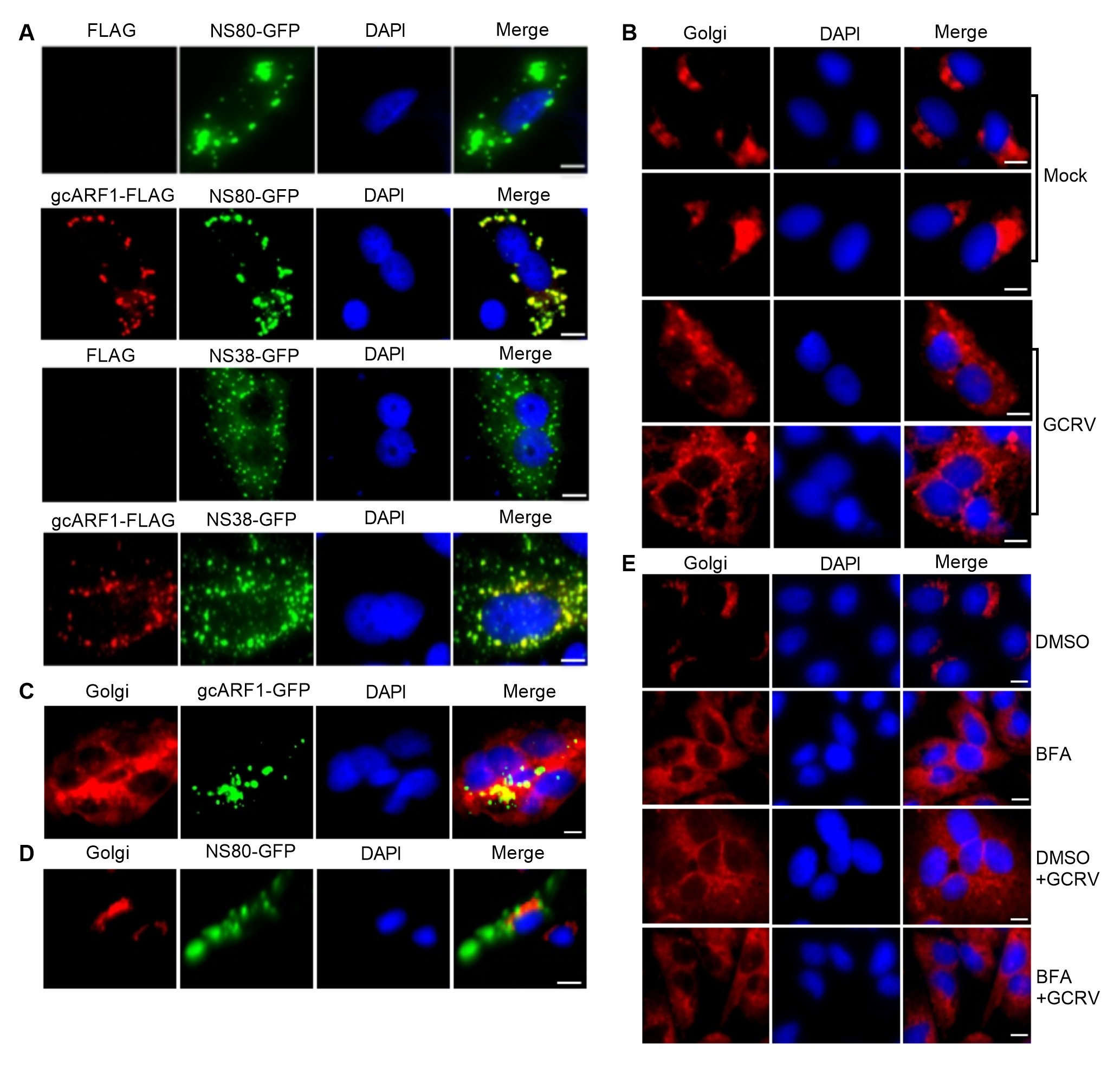

Supplement: Supplementary file 1 [file DataSheet_1.zip › Figure S2.tif]

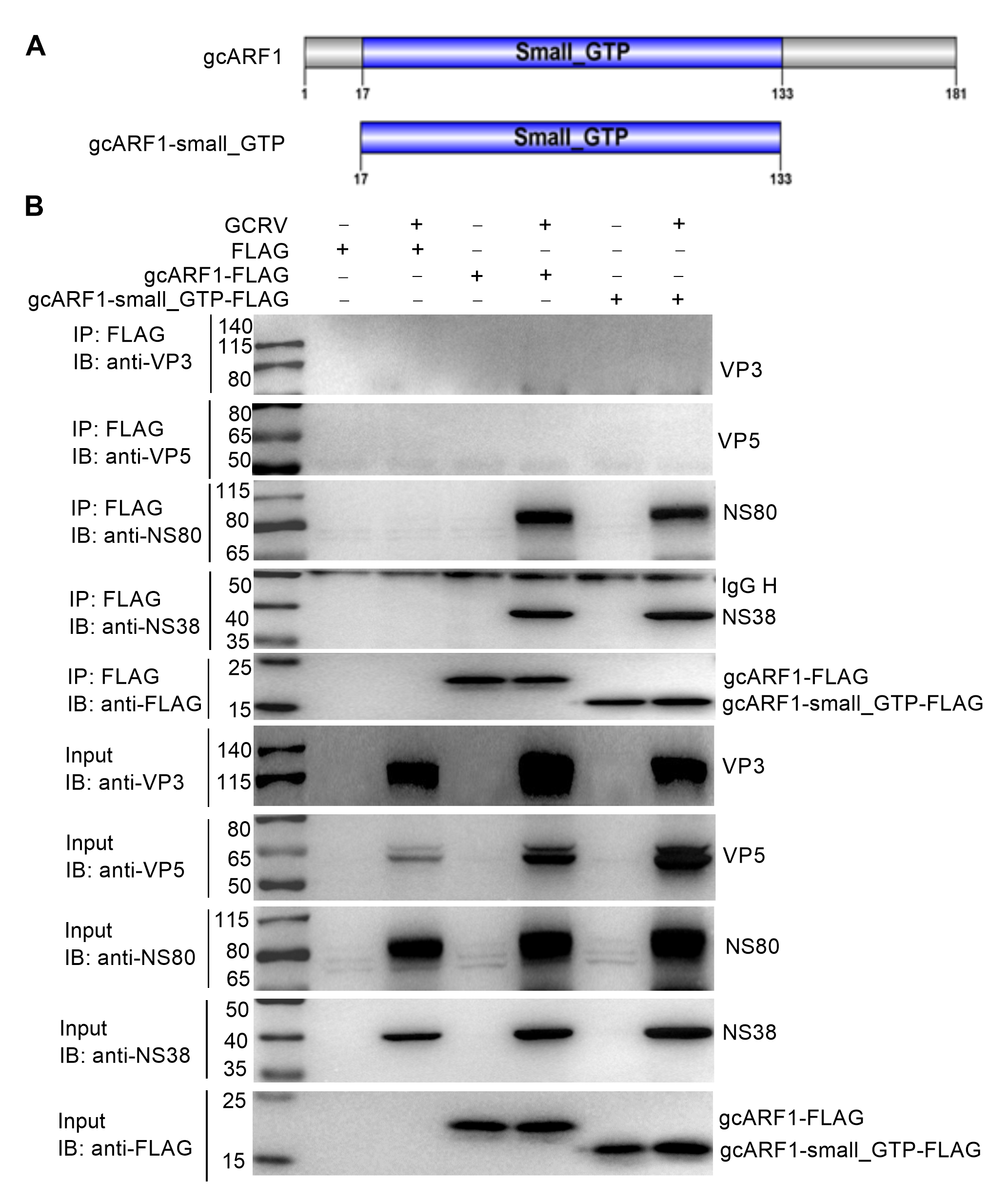

Supplement: Supplementary file 1 [file DataSheet_1.zip › Figure S3.tif]

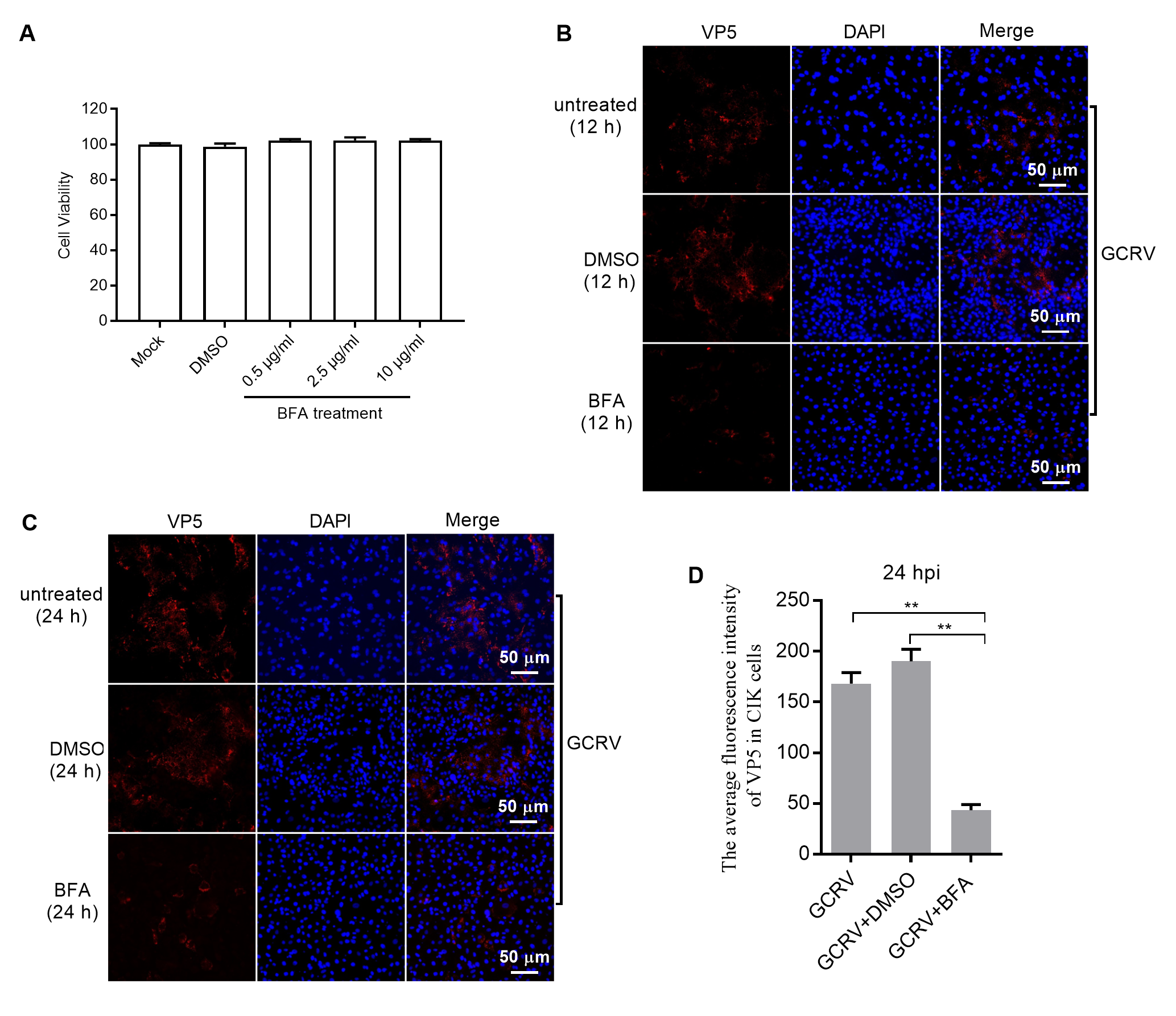

Supplement: Supplementary file 1 [file DataSheet_1.zip › Figure S4.tif]

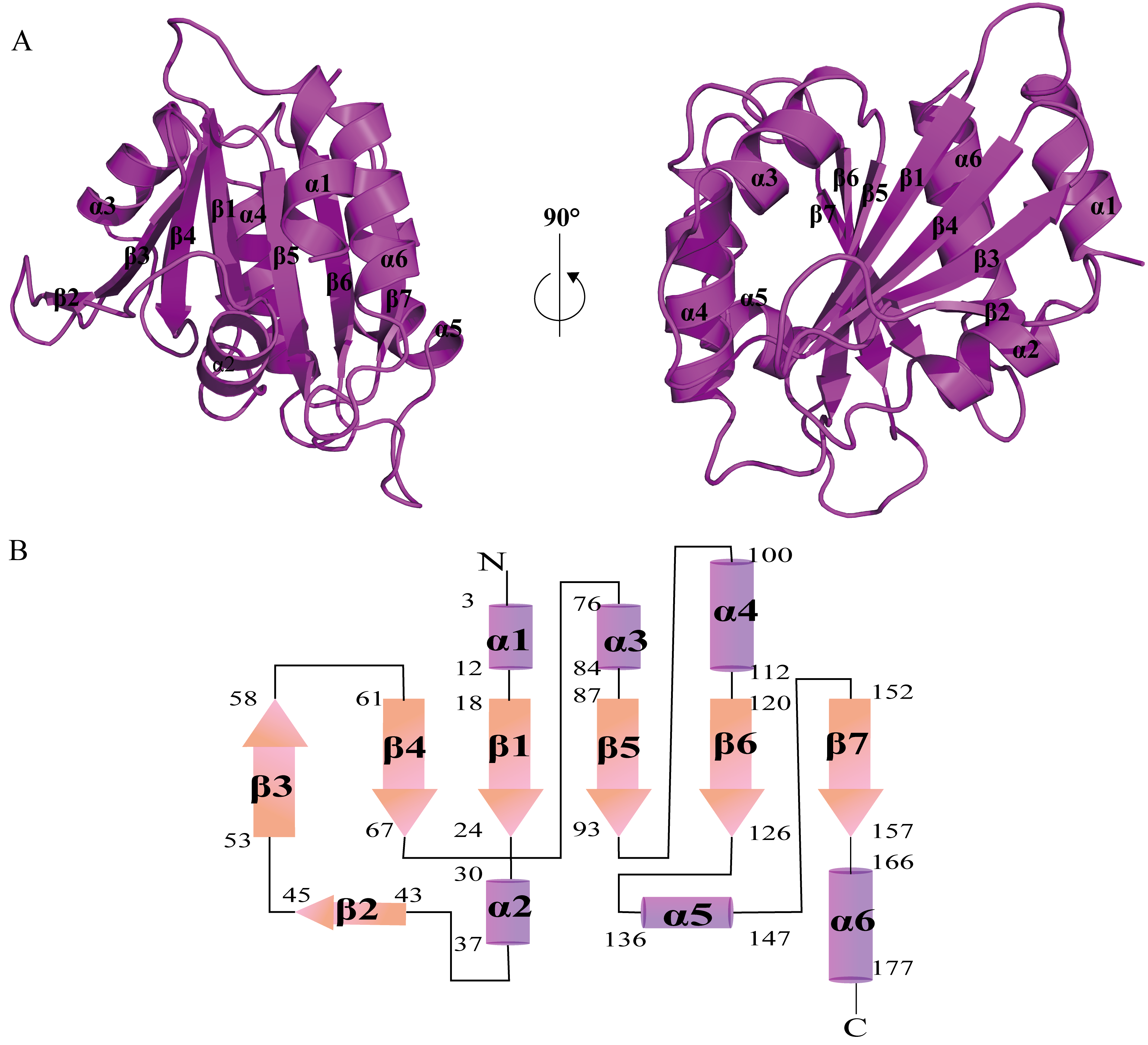

Supplement: Supplementary file 1 [file DataSheet_1.zip › Figure S5.tif]

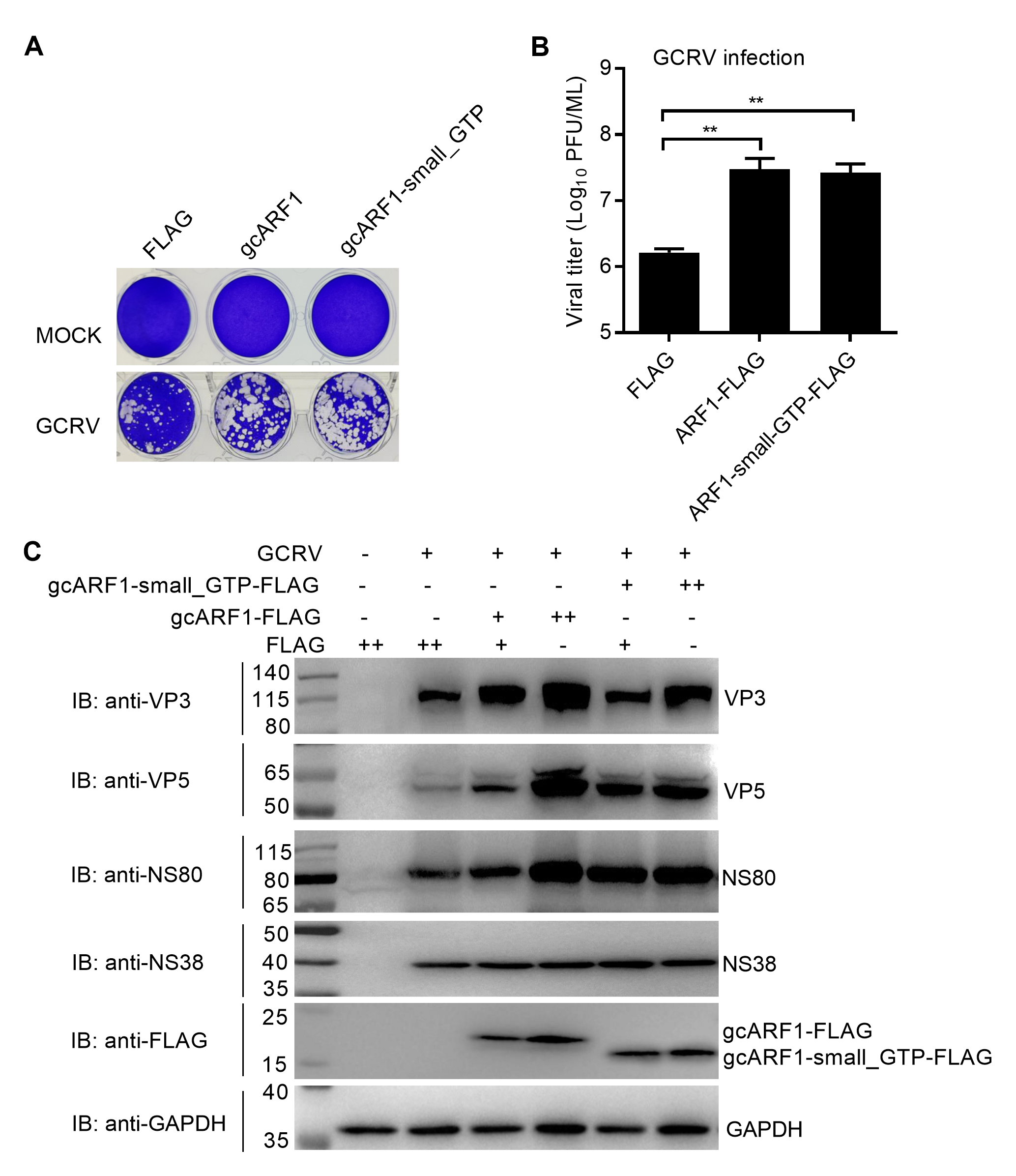

Supplement: Supplementary file 1 [file DataSheet_1.zip › Figure S6.tif]
